# Lateral Preoptic Area Neurons Activated by Angiotensin-(1–7) Increase Intravesical Pressure: A Novel Feature in Central Micturition Control

**DOI:** 10.3389/fphys.2021.682711

**Published:** 2021-07-12

**Authors:** Gustavo B. Lamy, Eduardo M. Cafarchio, Bárbara do Vale, Bruno B. Antonio, Daniel P. Venancio, Janaina S. de Souza, Rui M. Maciel, Gisele Giannocco, Patrik Aronsson, Monica A. Sato

**Affiliations:** ^1^Department of Morphology and Physiology, Faculdade de Medicina do ABC, Centro Universitario FMABC, Santo Andre, Brazil; ^2^Department of Medicine, Federal University of São Paulo, São Paulo, Brazil; ^3^Department of Biological Sciences, Federal University of São Paulo, Diadema, Brazil; ^4^Department of Pharmacology, Institute of Neuroscience and Physiology, Sahlgrenska Academy, University of Gothenburg, Gothenburg, Sweden

**Keywords:** intravesical pressure, lateral preoptic area, urinary bladder, ACE-2, mas receptor, micturition

## Abstract

Central micturition control and urine storage involve a multisynaptic neuronal circuit for the efferent control of the urinary bladder. Electrical stimulation of the lateral preoptic area (LPA) at the level of the decussation of the anterior commissure in cats evokes relaxation of the bladder, whereas ventral stimulation of LPA evokes vigorous contraction. Endogenous Angiotensin-(1–7) [(Ang-(1–7)] synthesis depends on ACE-2, and its actions on binding to Mas receptors, which were found in LPA neurons. We aimed to investigate the Ang-(1–7) actions into the LPA on intravesical pressure (IP) and cardiovascular parameters. The gene and protein expressions of Mas receptors and ACE-2 were also evaluated in the LPA. Angiotensin-(1–7) (5 nmol/μL) or A-779 (Mas receptor antagonist, 50 nmol/μL) was injected into the LPA in anesthetized female Wistar rats; and the IP, mean arterial pressure (MAP), heart rate (HR), and renal conductance (RC) were recorded for 30 min. Unilateral injection of Ang-(1–7) into the LPA increased IP (187.46 ± 37.23%) with peak response at ∼23–25-min post-injection and yielded no changes in MAP, HR, and RC. Unilateral or bilateral injections of A-779 into the LPA decreased IP (−15.88 ± 2.76 and −27.30 ± 3.40%, respectively) and elicited no changes in MAP, HR, and RC. The genes and the protein expression of Mas receptors and ACE-2 were found in the LPA. Therefore, the LPA is an important part of the circuit involved in the urinary bladder control, in which the Ang-(1–7) synthetized into the LPA activates Mas receptors for increasing the IP independent on changes in RC and cardiovascular parameters.

## Introduction

Urinary bladder dysfunctions can make the daily life activities difficult, causing social and mental discomfort. In the nephrology and urology outpatient care units, almost 40% of the patients present disorders of the lower urinary tract (Bakker et al., 2002; Kajiwara et al., 2004; Hashim et al., 2009; Sureshkumar et al., 2009). Among the urinary bladder dysfunctions, urinary incontinence symptoms have been reported with higher prevalence in women (Aoki et al., 2017).

Central control of micturition and urine storage involves a complex and not fully understood mechanism. The maintenance of excretion and urinary storage depends on reflex mechanisms; however, this reflex arc can undergo direct cortical influence through facilitatory and inhibitory mechanisms. The onset of micturition is facilitated by the Pontine Micturition Center (PMC) (de Groat, 1998), while urinary storage is influenced by the Pontine Urine Storage Center (PUSC), which is located ventrolaterally in PMC.

Studies carrying out the injection of pseudorabies virus into the urinary bladder wall have shown that, after a long incubation period, infected neurons are found in the lumbosacral spinal cord, raphe nucleus, reticular formation, pontine urination center (PMC), locus coeruleus, red nucleus, hypothalamus, preoptic area, and cortical areas (Nadelhaft et al., 1992). This evidence indicates that a multisynaptic neuronal circuit is involved in the efferent control of the urinary bladder.

The lateral preoptic area (LPA) is located in the hypothalamus and connects with limbic structures involved in physiological and behavioral responses to stress (Wayner et al., 1983; Briski and Gillen, 2001). The LPA also has osmosensitive neurons that regulate water intake (Osaka et al., 1995). Evidence suggests that LPA neurons would be important in the inhibitory control of water intake, as the chemical damage of this area with ibotenic acid increased water intake either upon administration of hypertonic saline or in water-deprived rats for 14 h (Saad et al., 1996). In addition, chemical lesions of the LPA using high doses of kainic acid have demonstrated that rats show polydipsia accompanied by increased urine production; however, this effect was reversed 1 week after lesions (Osaka et al., 1993).

Kabat et al. (1936) have shown that electrical stimulation in scattered points of the LPA at the level of the decussation of the anterior commissure in cats evoked relaxation of the bladder. Nevertheless, the stimulation of the ventral portion of LPA causes a strong bladder contraction. This portion of LPA contains fibers of the medial forebrain bundle (Kabat et al., 1936).

The discovery of Angiotensin-(1–7) [Ang-(1–7)] formed by anangiotensin-converting enzyme (ACE)-1-independent pathway, which binds to the Mas receptors *in vitro* and *in vivo*, allowed its recognition as a biologically active peptide of the Renin-Angiotensin System (Santos et al., 1988, 2005; Schiavone et al., 1988; Campagnole-Santos et al., 1989; Chappell et al., 1989; Carey and Siragy, 2003; Ferrario, 2006; Bader, 2010). Angiotensin-(1–7) can be synthetized by different pathways. One of them is through ACE-2-dependent cleavage of Angiotensin I in Angiotensin-(1–9), which, in turn, undergoes the action of ACE and subsequent hydrolysis by neutral endopeptidase 24.11 (NEP) (Chappell et al., 2000; Rice et al., 2004). The other pathway, which has been considered as the most physiologically relevant, is through the cleavage of Angiotensin II by ACE-2 in Ang-(1–7) (Vickers et al., 2002).

Evidence indicates that the ACE-2/Ang-(1–7)/Mas receptor axis is capable of promoting effects contrary to the harmful actions of the ACE-1/Angiotensin II/AT-1 receptor, especially in pathological conditions (Ferreira and Santos, 2005). Nevertheless, the functions of Ang-(1–7) are not limited to counter-regulatory actions (Santos et al., 2000). Studies with gene deletion of the Mas receptor lead to the appearance of several alterations; among which are cardiac dysfunction (Santos et al., 2006), increased blood pressure (Xu et al., 2008), decreased baroreflex function (de Moura et al., 2010), endothelial dysfunction (Xu et al., 2008), and also changes similar to the metabolic syndrome (Santos et al., 2006). Different areas in the medulla oblongata involved in cardiovascular control, as well as forebrain areas as the supraoptic nucleus and the LPA, showed the existence of neurons labeled for Mas receptors, using the immunofluorescence technique (Becker et al., 2007).

The prior studies in cats were performed, using electrical stimulation into the LPA, which can stimulate either cell bodies or axons from other brain areas. Earlier reports showed the Ang-(1–7) actions in some hypothalamic areas, and Mas receptors have been found in LPA. Nevertheless, to the best of our knowledge, no previous evidence showed whether Ang- (1–7) could act or not in the LPA to modulate the control of urinary bladder. Thereby, we hypothesized that Ang-(1–7) acts in LPA neurons, changing the intravesical pressure (IP) in female rats. In order to do that, we investigated the effects of Ang-(1–7) and A-779 (Mas receptor antagonist) injections into the LPA on the IP. In addition, the gene and protein expressions of Mas receptors and ACE-2 were evaluated in the LPA for demonstrating the existence of Ang-(1–7) receptors in the LPA neurons and also for understanding if Ang-(1–7) is produced in neurons located in LPA, respectively.

## Materials and Methods

### Animals

Female Wistar rats (∼230–250 g, 14–16 weeks old) supplied by the Animal Facility of the Faculdade de Medicina do ABC were used. The animals were initially housed in groups of four rats per plastic cage. After stereotaxic surgery for implantation of guide cannulas into the LPA, the rats were maintained in individual plastic cages and provided with standard chow pellets and tap water *ad libitum*. The humidity of the animal room was maintained at ∼70%, and the room temperature was controlled with an air conditioner set at 22–24°C with a 12:12-h light–dark cycle. All procedures were performed in accordance with the National Institutes of Health (NIH) Guide for the Care and Use of Laboratory Animals, and were approved by the Animal Ethics Committee of the Faculdade de Medicina do ABC, Centro Universitario FMABC (protocol number 13/2017).

### Implantation of Guide Cannulas Into the LPA

Rats were anesthetized with i.p. ketamine (50 mg/kg, Dopalen^®^, Ceva Saude Animal Ltda, Paulinia, Brazil) and i.m. xylazine (10 mg/kg, Anasedan^®^, Ceva Saude Animal Ltda, Paulinia, Brazil). They were placed in a stereotaxic apparatus (David Kopf, Tujunga, CA, United States), and the antisepsis in the surgical field was performed, using polyvinyl pyrrolidone. A midline incision was carried out in the skin on the skull to expose the bregma and lambda sutures that were positioned at the same horizontal plane. A stainless steel guide cannula (12-mm length, 23 gauge, 0.642-mm OD, 0.337-mm ID, BD, Juiz de Fora, Brazil) was implanted into the brain with the tip located.8-m caudal from bregma, ± 1.5 mm lateral from midline, and 7.2 mm ventral to the cranial surface at the anteroposterior level on the spot for insertion of the guide cannula. Two jeweler screws were implanted in the skull, and the guide cannula was anchored to the screws with acrylic cement. The guide cannula was closed, using a mandrel of 12-mm length with the external tip involved by a polyethylene tubing cap, which fitted to the guide cannula. At the end of the surgery, the rats received a single dose of i.m. Veterinary Pentabiotic for Small Animals (2,000 U/mL, 0.1 mL/rat, Fort Dodge Saude Animal, Campinas, Brazil) as a prophylactic procedure and i.m. meloxicam (0.2 mg/kg per day, Maxicam, OurofinoSaude Animal, Campinas, Brazil) for 3 days to produce postoperative analgesia and anti-inflammatory effect.

### Surgical Preparation for Cardiovascular Parameters and Intravesical Pressure Recordings

The rats were anesthetized with 2% isoflurane in 100% O_2_ and submitted to:

#### Cannulation of the Femoral Artery and Vein

Polyethylene tubing (PE-50 connected to PE-10, Clay Adams, NJ, United States) was inserted into the femoral artery and veinfor pulsatile arterial pressure (PAP), mean arterial pressure (MAP), and heart rate (HR) recordings in the data acquisition system (PowerLab 16 SP, AD Instruments, Castle Hill, AU), and for drug administration, respectively.

#### Measurement of Regional Blood Flow

A midline laparotomy was carried out in order to isolate the left renal artery, and a miniaturized pulsed Doppler flow probe (0.8 mm in diameter, Iowa Doppler Products, Iowa City, IA, United States) was placed around this artery for indirect measurement of the blood flow and renal conductance (RC). The probe was connected to a Doppler flow meter (Department of Bioengineering, The University of Iowa, Iowa City, IA, United States), and the amplified signal was digitalized in a data acquisition system (PowerLab 16 SP, AD Instruments, Castle Hill, AU). Additional details about the Doppler technique, including the readability of this method for estimation of the blood velocity, were previously described by Haywood et al. (1981). Relative renal vascular conductance was calculated as the ratio of Doppler shift (kHz) and mean arterial pressure (MAP, mmHg). The data were presented as percent change from the baseline [(final conductance–initial conductance)/initial conductance] × 100.

#### Cannulation of the Urinary Bladder

A small incision in the bladder wall was carried out for insertion of polyethylene tubing (PE-50 connected to PE-10, Clay Adams, NJ, United States), filled with saline at the apex of the bladder as previously described by Cafarchio et al. (2016, 2018, 2020) and Magaldi et al. (2020). A small drop of tissue glue was used to fix the catheter on the bladder wall for intravesical pressure (IP) recordings in a data acquisition system (PowerLab 16 SP, AD Instruments, Castle Hill, AU). The urethra outlet was not submitted to ligature in order to permit the bladder voiding if necessary. A baseline intravesical pressure (IP) value was set at ∼5–7 mmHg by saline infusion or urine withdrawal through the catheter inserted into the urinary bladder. Percent changes in intravesical pressure (%ΔIP) were calculated as [(peak IP response—baseline IP)/baseline IP] × 100.

### Microinjection of Drugs

Microinjections of drugs into the LPA were made with a needle (27 gauges, 0.413-mm O.D., 0.210-mm I.D., 13-mm length, Injex, São Paulo, Brazil) connected to a 10-μL Hamilton syringe (Reno, NV, United States) by polyethylene tubing (PE-10, Clay Adams, NJ, United States). The volume of all the drugs injected into the LPA was 1 μL.

### Histology

At the end of the experiments, the animals were deeply anesthetized with i.v. sodium thiopental (170 mg/kg, Cristalia, Itapira, Brazil), and a microinjection of 4% Chicago Sky Blue dye (Sigma Aldrich, St. Louis, MO, United States) in a volume of 1 μL was made through the guide cannula in order to determine the sites of drug injections. The animals were transcardially infused with 10% formalin solution (Synth, Diadema, Brazil). The brains were harvested and maintained in 10% formalin for at least 24 h and, thereafter, cut in 40-μm sections, using a freezing microtome (Leica Biosystems, Buffalo Grove, IL, United States), and stained with 2% neutral red (Sigma Aldrich, St. Louis, MO, United States). The slices were covered with Entellanmounting medium (Merck) and analyzed with a light field microscope (Nikon Eclipse E-200, Tokyo, Japan) to verify the presence of the Chicago Sky blue dye in the site of injection.

### Experimental Design

#### Effects of Ang-(1–7) and A-779 Into the LPA on Intravesical Pressure, Arterial Pressure, Heart Rate, and Renal Conductance in the Female Rats (*n* = 6/group)

**FIGURE 1:**
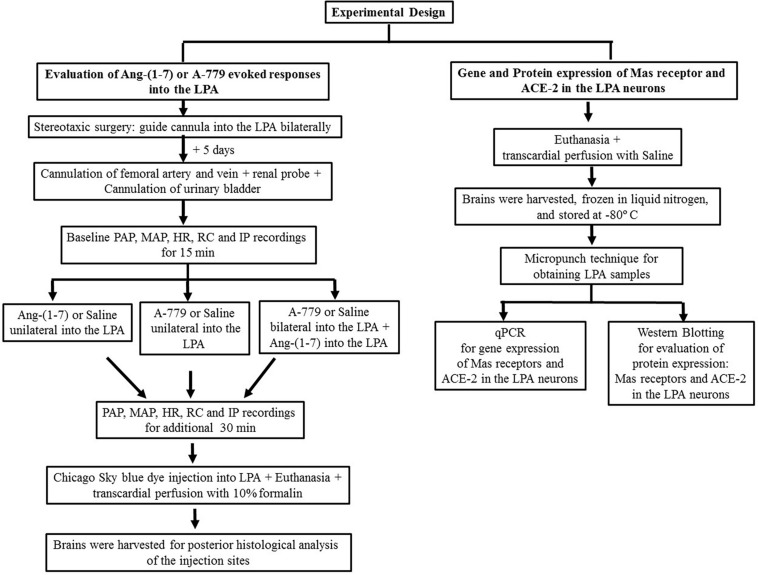
A flowchart depicting the experimental design details.

**FIGURE 2:**
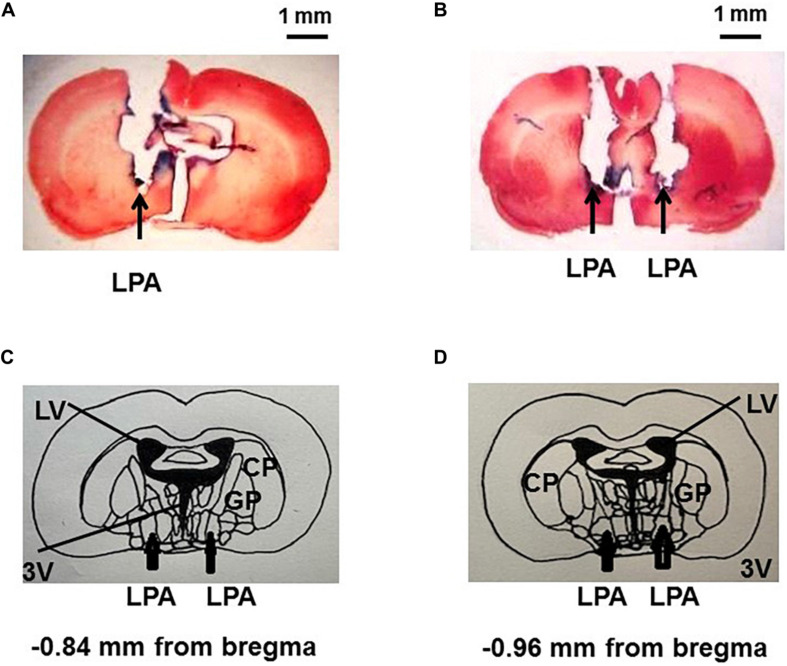
Photomicrograph of histological sections (coronal), showing the drug injection site into the lateral preoptic area unilaterally **(A)** and bilaterally **(B)** marked with 4% Chicago Sky Blue dye (1 μL). A schematic representation of the lateral preoptic area at –0.84 mm from bregma **(C)** and at –0.96 mm from bregma **(D)** is shown in the brain sections according to Paxinos and Watson (2009). The arrows indicate the location of the LPA. CP, caudate nucleus/putamen; GP, globus pallidus; LPA, lateral preoptic area; LV, lateral ventricle; SFO, subfornical organ; 3V, third brain ventricle. Amplification: 160×.

As shown in Figure 1, first of all, the rats under ketamine + xylazine anesthesia underwent an implant of a guide cannula into the LPA, using a stereotaxic apparatus. Five days later, after recovery of the stereotaxic surgery, the animals were anesthetized with 2% isoflurane in 100% O_2_ and submitted to the cannulation of the femoral artery and vein for PAP, MAP, and HR recordings, and infusion of drugs, respectively. A miniaturized Doppler flow probe was placed around the left renal artery for indirect blood flow measurement. Polyethylene tubing was also inserted into the urinary bladder for IP recordings. Rectal temperature was maintained between 37 and 38°C, using a heating pad. The animals were anesthetized with 2% isoflurane in 100% O_2_ during the whole experiment and were unresponsive to a noxious toe pinch. This experimental approach was carried out as previously reported by Cafarchio et al. (2016, 2018, 2020) and Magaldi et al. (2020). A steady level of arterial pressure was maintained under anesthesia. The rats were kept in supine position in order to avoid pressure of abdominal organs on the urinary bladder, which could affect the IP values. After baseline PAP, MAP, HR, renal blood flow, and IP recordings for 15 min, Ang-(1–7) (5 nmol/μL, catalog # A9202, Sigma Aldrich, St, MO, United States) or saline (vehicle, 1 μL) or A-779 trifluoroacetate salt (50 nmol/μL, a Mas receptor antagonist, catalog # SML1370, Sigma Aldrich, St, MO, United States) was injected into the LPA unilaterally, and all the parameters were recorded for additional 30 min. In another group of the rats, after the baseline recordings, saline (vehicle, 1 μL) or A-779 (50 nmol/μL, 1 μL) was injected bilaterally into the LPA, and the effectiveness of Mas receptor blockade was evaluated by Ang-(1–7) injections (5 nmol/μL) bilaterally into the LPA at 15-min post-A-779 injections into the LPA, and all the parameters were recorded for at least 30 min. At the end of the experiment, a 4% Chicago Sky blue dye (1 μL) was administrated in the injection sites. An overdose of sodium thiopental (170 mg/kg, i.v.) was used to euthanize the animals. The brains were removed for posterior histological evaluation. Figure 2 shows the site of dye deposition in the LPA. Only the animals with histological confirmation of microinjection sites in the LPA were considered in this study.

#### Gene Expression of Mas Receptors and ACE-2 in the LPA Neurons (*n* = 6)

As depicted in Figure 1, the animals were deeply anesthetized with isoflurane 4% in O_2_ 100% and submitted to a thoracotomy for transcardial perfusion of 40 mL of saline. After that, the skull bone was removed, using a rongeur (WPI Instruments, Sarasota, FL, United States), and the brain was harvested with forceps and immediately frozen in liquid nitrogen and stored at −80°C in an ultrafreezer until the day of total RNA extraction with the TRizol^®^ reagent. To obtain LPA samples, the brain was sliced and a micropunch was performed on the frozen sections of rat brain. The animals used in this protocol were not previously instrumented for cardiovascular recordings. The procedures for gene expression of Mas receptors, ACE-2, and cyclophilin A were performed by quantitative real-time polymerase chain reaction (qPCR) as follows:

Total RNA was isolated from frozen LPA samples with TRIzol Reagent^®^ (Thermo Fisher Scientific) according to the protocol of the manufacturer. RNA integrity was checked by agarose gel electrophoresis, and RNA purity reached the following criteria: A260/280 ≥ 1.8. The extracted total RNA concentration was measured, using a NanoDrop^TM^ (One-One c) spectrophotometer (Thermo Fisher Scientific), and 1 μg of total RNA was subjected to reverse transcription reaction. Complementary DNA (cDNA) synthesis was generated, using ImProm-II^TM^ Reverse Transcription System (Promega, Madison, WC, United States) according to the protocol of the manufacturer. Quantitative real-time PCR (qPCR) was carried out, using 2 μL of cDNA and the EvaGreen^TM^ qPCR Mix Plus (Solis BioDyne, Tartu, Estonia) in the ABI Prism 7500 Sequence Detection System (Applied Biosystems, Foster City, CA, United States) to amplify specific primers sequences for the Mas receptors, ACE-2, and cyclophilin A. The forward and reverse primers sequences (Thermo Fisher Scientific) for rats used in this study follow below:

Mas receptor:

(forward) 5′-CCTGCATACTGGGAAGACCA-3′

(reverse) 5′-TCCCTTCCTGTTTCTCATGG-3′

ACE-2:

(forward) 5′-TTGAACCAGGATTGGACGAAA-3′

(reverse) 5′-GCCCAGAGCCTACGATTGTAGT-3′

Cyclophilin A:

(forward)—5′-CCCACCGTGTTCTTCGACAT-3′

(reverse)—5′-CTGTCTTTGGAACTTTGTCTGCAA-3′

Cyclophilin A was used as internal control (a housekeeping gene). The procedure consisted of an initial step of 10 min at 95 C, followed by 45 cycles of 20 s each at 95 C, 20 s at 58 C, and 20 s at 72 C. Gene expression was determined by CT, and all values were expressed, using cyclophilin A mRNA as an internal control.

#### Protein Expression of the Mas Receptor and ACE-2 in LPA Neurons (*n* = 6)

A different group of rats (from that used to gene expression, according to the approach described in Figure 1) was deeply anesthetized with isoflurane 4% in 100% O_2_ and underwent a thoracotomy for transcardial perfusion of saline. Afterward, the skull bone was removed, using a rongeur (WPI Instruments, Sarasota, FL, United States), and the brain was removed with forceps and immediately frozen in liquid nitrogen and stored at −80°C in an ultrafreezer for later determination of protein expression of the Mas receptor and ACE-2 in the LPA neurons by Western Blotting. The rats used in this protocol were not animals previously instrumented for cardiovascular recordings. The procedures for Western Blotting assay were carried out as follows:

The samples from the LPA were placed in RIPA lysis and an extraction buffer, added with a mixture of protease and phosphatase inhibitors (Thermo Fisher Scientific). The tissues were homogenized in a lysis buffer, incubated on ice for 10 min, and centrifuged at 7,000 *g* for 5 min at 4°C, and the supernatant containing the soluble proteins was stored at −80°C. The protein concentration was determined, using NanoDrop^TM^ (One-One c) spectrophotometer (Thermo Fisher Scientific). The total proteins were separated on a 10% SDS-acrylamide gel and then transferred electrophoretically to the nitrocellulose membrane (Bio-rad), using the *Trans-*blot Turbo Transfer device (Bio-rad). The membrane was stained with Ponceau solution to check successful transfer. The membrane was photographed in the Chemidoc device (Biorad) for determination of total protein by densitometry, using the Image Lab^TM^ software (Bio-rad). After that, the membrane was washed with milli-Q water at least three times and then incubated for 1 h with 5% non-fat milk in Tris-buffered saline −0.1% Tween 20 (TBS-T). After this period, this solution was discarded, and the membrane was incubated at 4°C overnight, with a polyclonal primary antibody specific for the Mas receptor (rabbit anti-Mas, Novus Biologicals, catalog # NBP1-78444) and for ACE-2 (rabbit anti-ACE-2, Cloud-Clone Corp., catalog # PAB886Ra01) diluted to a concentration of 1:250 in TBS-T. The blots were washed with TBS-T and then incubated with a goat-anti-rabbit secondary antibody (Alexa Fluor 488, ThermoFischer Scientific) in a 1:10,000 dilution for 1 h, which produced a chemiluminescent reaction. After that, the membrane was filmed in the Chemidoc device (Bio-rad). The blot corresponding to the protein of interest was quantified by densitometry, using the Image Lab^TM^ software (Bio-rad). The optical density (O.D.) of the proteins of interest was normalized by the expression of total protein.

### Statistics

A Kolmogorov–Smirnov test for normality was used to verify the data distribution. Results fit to a normal distribution were expressed as a mean ± S.E.M. The data were submitted to unpaired Student’s *T*-tests for comparison between the %ΔIP or %ΔRC responses evoked by Ang-(1–7) or A-779 versus saline into the LPA. Paired Student’s *T*-tests were used to compare MAP and HR before and after Ang-(1–7) or A-779 or saline into the LPA. Statistical analysis was conducted, using the statistical software package Sigma Stat 3.5. The significance level was set at *P* < 0.05.

## Results

### Responses Elicited by Unilateral Injection of Ang-(1–7) Into the LPA on Intravesical Pressure, Arterial Pressure, Heart Rate, and Renal Conductance in the Female Rats (*n* = 6)

**FIGURE 3:**
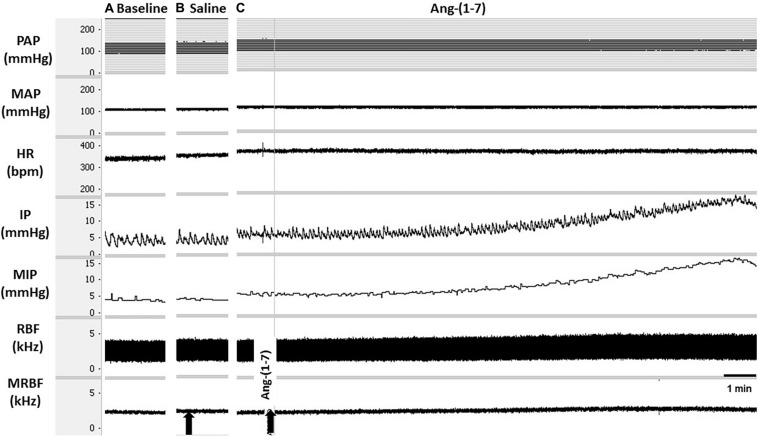
Tracings showing **(A)** the baseline pulsatile arterial pressure (PAP, mmHg), mean arterial pressure (MAP, mmHg), heart rate (HR, bpm), intravesical pressure (IP, mmHg), mean intravesical pressure (MIP, mmHg), renal blood flow (RBF, kHz), and mean renal blood flow (MRBF, kHz), and the responses evoked by **(B)** unilateral injection of saline (vehicle, 1 μL) or **(C)** angiotensin-(1–7) [Ang(1–7)] (5 nmol/μL, 1 μL) into the lateral preoptic area (LPA). Arrows indicate the moment of saline or Ang-(1–7) injection into the LPA.

At baseline [before injections of saline or Ang-(1–7) into the LPA], the MAP was 96 ± 3 mmHg, the HR was 344 ± 20 bpm, and the IP was 7.12 ± 0.76 mmHg (*n* = 6) (Figure 3A).

**FIGURE 4:**
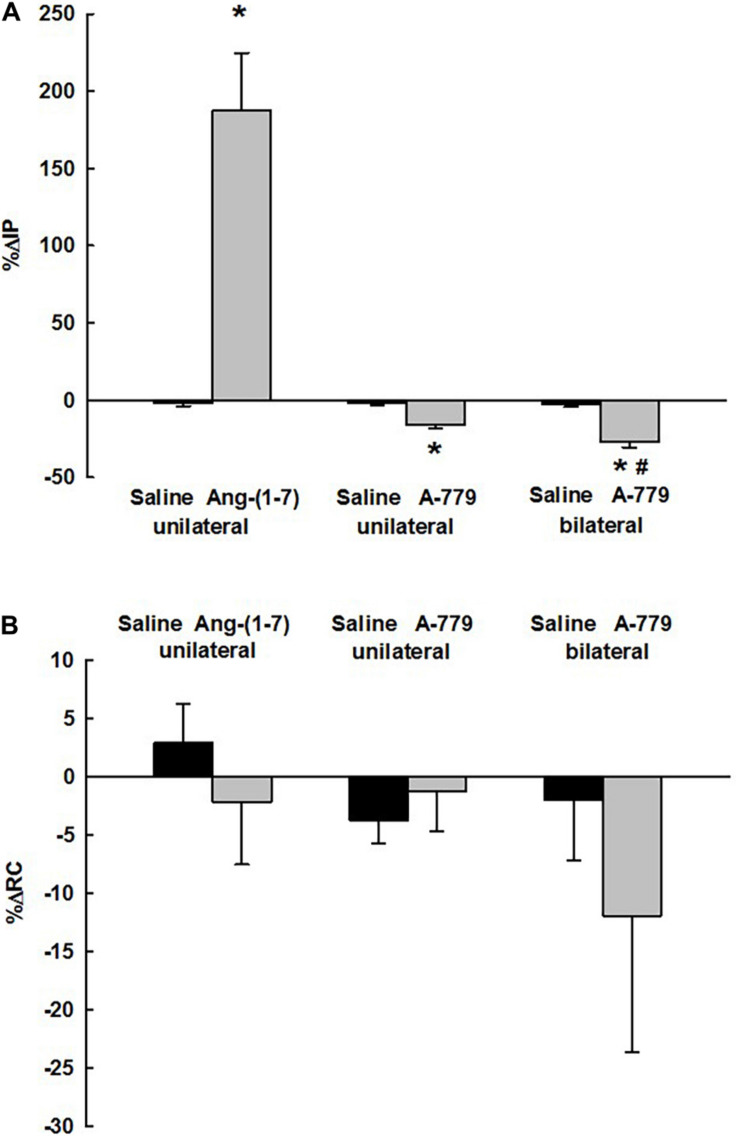
**(A)** Percent changes in intravesical pressure (%ΔIP) and **(B)** percent changes in renal conductance (%ΔRC) in three different groups of rats (*n* = 6/group) evoked by unilateral injection of saline (vehicle, 1 μL) or angiotensin-(1–7) [Ang(1–7)] (5 nmol/μL, 1 μL) into the lateral preoptic area (LPA); unilateral injection of saline or A-779 (50 nmol/μL, Mas receptor antagonist, 1 μL) into LPA; bilateral injections of saline or A-779 (50 nmol/μL, Mas receptor antagonist, 1 μL) into LPA. *P < 0.05 vs. saline, ^#^*P* < 0.05 vs. unilateral A-779 into the LPA.

The unilateral injection of saline (vehicle) into the LPA yielded no significant changes in MAP (−2 ± 1 mmHg), HR (1 ± 2 bpm), RC (2.90 ± 3.34%), and IP (−2.16 ± 1.94%) compared with baseline values (Figure 3B). Although the unilateral injection of Ang- (1–7) into the LPA produced no significant changes in MAP (2 ± 2 mmHg), HR (–7 ± 7 bpm), and RC (−2.23 ± 5.30%), a significant marked increase in IP (187.46 ± 37.23%) was observed, both compared with baseline parameters and in comparison with saline injection into the LPA (*p* < 0.05) (Figures 3C, 4). The latency for the onset of IP increase evoked by Ang-(1–7) administrated into the LPA was ∼10 min, and the peak response was achieved at ∼23–25 min after the injection (Figure 3).

### Responses Evoked by Unilateral Injection of A-779 (Mas Receptor Antagonist) Into the LPA on Intravesical Pressure, Arterial Pressure, Heart Rate, and Renal Conductance in the Female Rats (*n* = 6)

**FIGURE 5:**
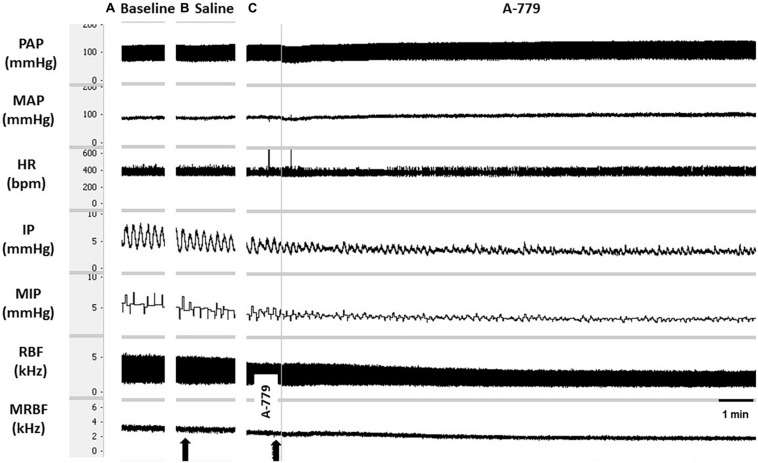
Tracings showing **(A)** the baseline pulsatile arterial pressure (PAP, mmHg), mean arterial pressure (MAP, mmHg), heart rate (HR, bpm), intravesical pressure (IP, mmHg), mean intravesical pressure (MIP, mmHg), renal blood flow (RBF, kHz), and mean renal blood flow (MRBF, kHz), and the responses evoked by **(B)** unilateral injection of saline (vehicle, 1 μL) or **(C)** A-779 (50 nmol/μL, Mas receptor antagonist, 1 μL) into the lateral preoptic area (LPA). Arrows indicate the moment of saline or A-779 injection into the LPA.

At the baseline (prior to injections of saline or A-779 into the LPA), the MAP was 90 ± 2 mmHg, the HR was 389 ± 12 bpm, and the IP was 6.47 ± 0.45 mmHg (*n* = 6) (Figure 5A).

The unilateral injection of saline (vehicle) into the LPA elicited no significant changes in MAP (1 ± 1 mmHg), HR (−4 ± 1 bpm), RC (−3.74 ± 2.02%), and IP (−1.95 ± 1.06%) compared with baseline parameters (Figure 5B). In contrast to Ang-(1–7) injections, we observed that the unilateral injection of A-779 into the LPA significantly decreased the intravesical pressure (−15.88 ± 2.76%), both compared with baseline and saline injections into the LPA (*p* < 0.05). However, the MAP (−1 ± 1 mmHg), HR (−5 ± 2 bpm), and RC (−1.30 ± 3.43%) showed no significant change compared with the baseline values and also in comparison to the saline injection into the LPA (Figures 5C, 4). The latency for the onset of IP decrease induced by unilateral injection of A-779 into the LPA was ∼10 min, and the peak response was achieved at ∼23–25 min after the injection (Figure 5).

### Responses Elicited by Bilateral Injections of A-779 Into the LPA on Intravesical Pressure, Arterial Pressure, Heart Rate, and Renal Conductance in Female Rats (*n* = 6)

Considering that the blockade of Mas receptors unilaterally in the LPA even in the absence of agonist produced a small reduction of intravesical pressure, another experimental group was performed, in which the guide cannulas were implanted bilaterally into the LPA in order to understand if any control could be exerted by the contralateral LPA neurons on intravesical pressure and also on the cardiovascular parameters.

**FIGURE 6:**
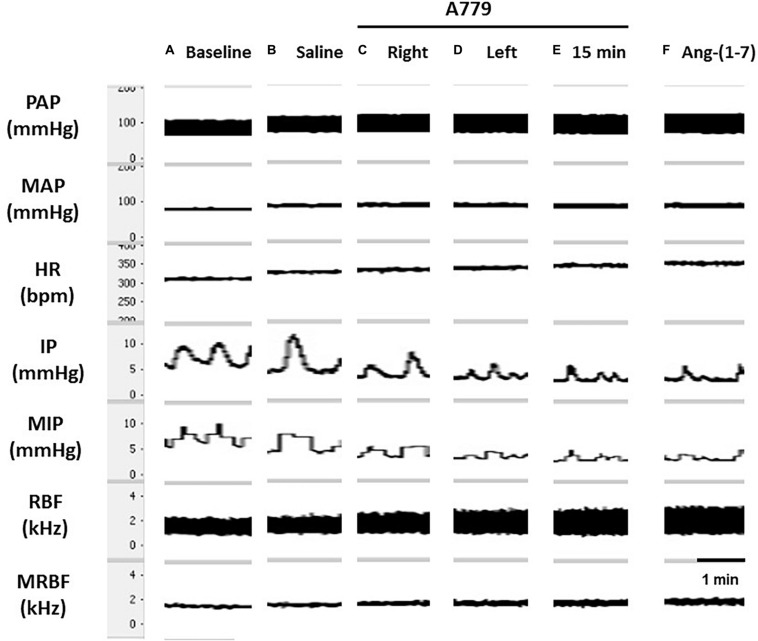
Tracings showing **(A)** the baseline pulsatile arterial pressure (PAP, mmHg), mean arterial pressure (MAP, mmHg), heart rate (HR, bpm), intravesical pressure (IP, mmHg), mean intravesical pressure (MIP, mmHg), renal blood flow (RBF, kHz), and mean renal blood flow (MRBF, kHz), and the responses evoked by **(B)** bilateral injections of saline (vehicle, 1 μL) or **(C–E)** A-779 (50 nmol/μL, Mas receptor antagonist, 1 μL) into the lateral preoptic area (LPA). The effectiveness of the Mas receptor blockade was also evaluated by **(F)** bilateral injections of angiotensin-(1–7) [Ang(1–7)] (5 nmol/μL, 1 μL) into the LPA for15 min after A-779 (50 nmol/μL, 1 μL).

In this group of rats, MAP was 91 ± 4 mmHg, HR was 340 ± 14 bpm, and IP was 7.16 ± 0.47 at the baseline (Figure 6A). Bilateral injections of saline into the LPA produced no significant changes in MAP (−2 ± 2 mmHg), HR (−3 ± 2 bpm), RC (−2.95 ± 5.32%), and IP (0.65 ± 6.41%) compared with baseline values (Figure 6B). After bilateral injections of A-779 into the LPA, a significant decrease in IP was observed (−27.30 ± 3.40% for 15 min after both injections) compared with the baseline and also in comparison with bilateral injections of saline into the LPA (*p* < 0.05). The decrease in IP evoked by bilateral injections of A-779 into the LPA was significantly greater (∼1.7-fold) than the reduction induced by the unilateral injection (−15.88 ± 2.76%, *p* = 0.026, unpaired Student’s *T*-test). No significant changes were observed in MAP (1 ± 1 mmHg), HR (1 ± 13 bpm) and RC (-11.97 ± 11.65%) after A-779 injected bilaterally into the LPA compared with the baseline and in comparison with the bilateral injections of saline into the LPA (Figures 6C–6E, 4).

The effectiveness of the Mas receptor blockade was further evaluated by the bilateral injections of Ang-(1–7) 15 min after the bilateral administration of A-779 into the LPA. The responses of Ang-(1–7) previously observed were abolished after a Mas receptor blockade as follows: %ΔIP (1.14 ± 2.66%), ΔMAP (−1 ± 0.3 mmHg), ΔHR (−2 ± 6 bpm), and %ΔRC (−2.35 ± 4.38%) compared with preinjection values (Figure 6F).

### Gene Expression of the Mas Receptors and ACE-2 in the LPA Neurons (*n* = 6)

**FIGURE 7:**
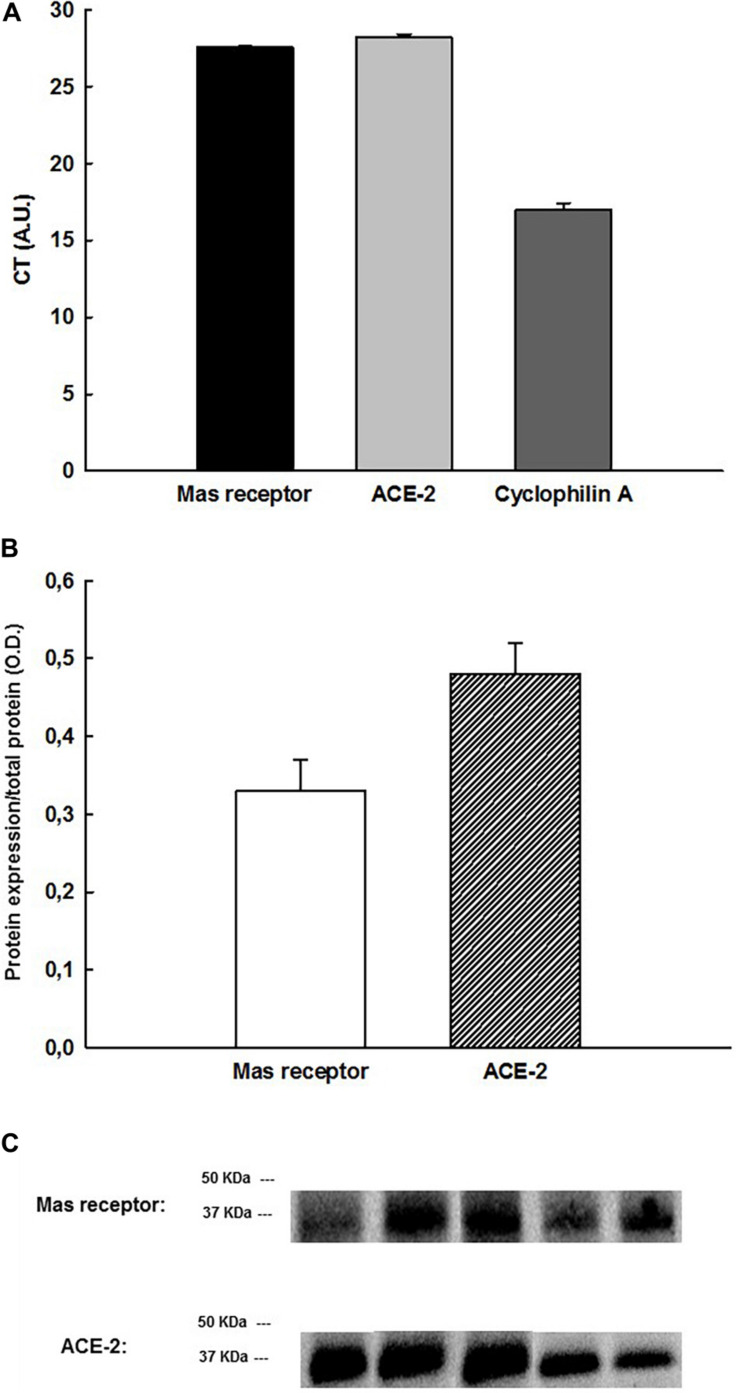
**(A)** CT values obtained by qPCR, showing the gene expression of Mas receptors, ACE-2 and cyclophilin A (housekeeping gene) in the LPA neurons (*n* = 6), **(B)** protein expression of Mas receptors and ACE-2 normalized by total protein in the LPA neurons by Western blotting (*n* = 6), **(C)** blots labeled for Mas receptors and ACE-2 in LPA samples by Western blotting.

The gene expression by qPCR demonstrated that the Mas receptor (CT = 27.60 ± 0.04 arbitrary units, A.U.), ACE-2 (CT = 28.22 ± 0.18 A.U.), as well as the housekeeping gene cyclophilin A (17.01 ± 0.42 A.U.), are present in the LPA samples (*N* = 6) (Figure 7A).

### Protein Expression of Mas Receptors and ACE-2 Gene in the LPA Neurons (*n* = 6)

The protein expression by Western Blotting showed the existence of Mas receptor (0.33 ± 0.04 O.D.) and ACE-2 (0.48 ± 0.04 O.D.) in LPA samples (*N* = 6) (Figures 7B,C).

## Discussion

The results of this study demonstrated that injections of Ang-(1–7) into the LPA evoked a marked increase in intravesical pressure compared with saline, whereas the blockade of Mas receptors for Ang-(1–7) with A-779 decreased intravesical both uni- or bilaterally compared with saline injections. However, it is noteworthy that bilateral injections of A-779 produced a synergistic effect compared with the unilateral injection. Despite the changes in intravesical pressure, no changes were observed in renal conductance, arterial pressure, and heart rate. Those findings suggest that the increases in intravesical pressure are not dependent on increases in urinary volume due to increases in the glomerular filtration rate. Although the unilateral blockade of Mas receptor into the LPA has yielded a slight but statistically significant decrease in intravesical pressure compared with saline injection, the bilateral injection of A-779 into the LPA enhanced the reduction in intravesical pressure. This suggests that neurons containing Mas receptors contralaterally into the LPA are tonically active and influence the control of the detrusor muscle tonus.

Previous studies of Kabat et al. (1936) showed that electrical stimulation of the LPA at the decussation level of the anterior commissure in cats evoked relaxation of the bladder. In the current study, the sites of injections into the LPA were located caudal from the anterior commissure level, which suggests that a different population of neurons has been activated by Ang-(1–7) injections into the LPA from extending .80- to .96-mm caudal from bregma (Paxinos and Watson, 2009). Kabat et al. (1936) also performed the stimulation of the ventral portion of LPA producing a strong bladder contraction. This portion of LPA contains fibers of the medial forebrain bundle (Kabat et al., 1936). It is not unlikely that the electrical stimulation performed in the study of Kabat et al. (1936) has also activated the cell bodies of neurons in the LPA, which could be the same population of neurons that underwent activation by Ang-(1–7) in the current study and increased the intravesical pressure.

Studies of Cafarchio et al. (2018) have demonstrated that the blockade of V1a and V2 receptors evokes a long-lasting decrease in intravesical pressure, suggesting that vasopressin is important for the maintenance of the detrusor muscle tonus. Both neural (de Groat, 1998) and humoral mechanisms (Cafarchio et al., 2018, 2020) seem to be involved in the control of the urinary bladder tonus. The increases in intravesical pressure induced by Ang-(1–7) showed a latency of roughly 10 min, and the peak response was observed at 23–25 min after injections into the LPA. This response with a delay for achieving the maximum response does not seem to be dependent on activation of autonomic efferents, and, instead of that, a humoral-dependent mechanism could be more likely involved in the development of the response evoked by Ang-(1–7) into the LPA. Earlier studies showed that LPA sends projections to the perinuclear shell of paraventricular nucleus of the hypothalamus, but a direct projection to magnocellular neurons responsible for vasopressin synthesis has not been demonstrated (Larsen et al., 1994). In contrast, *in vitro* studies performed in hypothalamic slices have shown that the electrical stimulation of cholinergic neurons in the LPA produces synaptic activation of vasopressin-synthesizing neurons in the supraoptic nucleus, which is blocked by hexamethonium but not by atropine (Hatton et al., 1983). Despite no previous study showed that Mas receptors-containing neurons synapse with cholinergic neurons in the LPA, the hypothesis that such interaction could be involved in the LPA in order to increase the intravesical pressure *via* vasopressin release should not be refused. In the current study, we have not measured the plasma vasopressin release after Ang-(1–7) into the LPA, which is a limitation of this study and requires further investigation.

The dose of A-779 used for the blockade of Mas receptors (50 nmol/μL or 50 mM) in the present study was 10-fold higher than the dose of Ang-(1–7) (5 nmol/μL or 5 mM). Nevertheless, we have observed a decrease in intravesical pressure that was not great as much as the increase in intravesical pressure evoked by Ang-(1–7) injected into the LPA. This likely happened because LPA should be one of the brain areas responsible for the maintenance of the urinary bladder tonus, and, upon the blockade of LPA neurons, other brain areas still provide the excitatory drive to the efferent pathways involved in the control of the detrusor muscle tonus. The decrease in intravesical pressure induced by the blockade of Mas receptors into the LPA also showed a latency of approximately 10 min, and the peak response was observed at 23–25 min after bilateral injections into the LPA. This pattern of response is suggestive of a tonic control played by both sides of the LPA that could be inhibiting the release of any humoral factor as vasopressin and, consequently, reducing the intravesical pressure. However, it is noteworthy that other brain areas involved in urinary bladder control as the cholinergic neurons in the medulla oblongata can also increase the plasma vasopressin release for rising the intravesical pressure at ∼30 min after carbachol injections into the 4th brain ventricle (4thV) (Cafarchio et al., 2016). The blockade of cholinergic receptors with atropine into the 4thV yields a decrease in intravesical pressure at 30 min after injections (Cafarchio et al., 2016), which shows similarities to the responses observed with A-779 injections bilaterally into the LPA, except by the differences in the latency for the onset of the responses.

In the current study, we have demonstrated the existence of Mas receptor and ACE-2 genes into the LPA by qPCR and also the protein expression of them by Western Blotting in LPA neurons. Angiotensin-(1–7) can be formed by different pathways. One of them is dependent on cleavage of Angiotensin I in Angiotensin-(1–9), which undergoes the action of ACE and subsequent hydrolysis by NEP (Chappell et al., 2000; Rice et al., 2004). The other pathway is through the cleavage of Angiotensin II by ACE-2 in Ang-(1–7), which has been the most suggested as the most relevant physiologically and biochemically (Vickers et al., 2002). Despite studies using the immunofluorescence assays in the central nervous system of Wistar rats have shown the presence of the Mas receptor in the lateral preoptic area (LPA) (Becker et al., 2007), no previous study demonstrated the gene expression by qPCR and protein expression by Western Blotting of the Mas receptors or ACE-2 in the LPA. Indeed, our findings suggest that the increases in intravesical pressure elicited by Ang-(1–7) are due to binding in the Mas receptors in LPA neurons, and the presence of ACE-2 in the LPA neurons suggests that Ang-(1–7) is endogenously synthetized by the neurons in this area.

Previous reports showed that lesions of the LPA neurons with ibotenic acid increased water intake upon administration of hypertonic saline or in water-deprivated rats for 14 h, suggesting a role of LPA in the inhibitory control of water intake (Saad et al., 1996). Furthermore, chemical lesions of the LPA, using high doses of kainic acid, have demonstrated that rats develop polydipsia accompanied by increased urine production; however, this effect was reversed 1 week after lesions (Osaka et al., 1993). In the current study, we have demonstrated that Ang-(1–7) neurons in the LPA are involved in the urinary bladder control; thereby, the LPA can be deemed as an important forebrain area of integrative mechanisms of hydroelectrolytic control and urinary bladder regulation; nevertheless, the physiological conditions in which the LPA mediates this integration still require further investigation.

In conclusion, our findings suggest that the LPA is an important part of the circuit involved in urinary bladder control, in which the Ang-(1–7) released into the LPA binds to the Mas receptors for increasing the intravesical pressure independent on changes in renal conductance and cardiovascular parameters. Our data are also suggestive that the neurons containing the Mas receptors bilaterally into the LPA are tonically active in the circuitry involved in the regulation of urinary bladder tone.

## Data Availability Statement

The raw data supporting the conclusions of this article will be made available by the authors, without undue reservation.

## Ethics Statement

The animal study was reviewed and approved by Comissão de Ética no Uso de Animais (CEUA-FMABC).

## Author Contributions

GBL carried out the functional experiments. JSS, RMM, and GG were responsible for the qPCR experiments. GBL, BV, BBA, DPV, and MAS performed the Western Blotting experiments. GBL, EMC, BA, DPV, and PA worked on data analysis and discussion. MAS designed the experiments, performed the statistical analysis, obtained the research grant, and also wrote the manuscript with GBL and PA. All the authors equally contributed to the development of the manuscript.

## Conflict of Interest

The authors declare that the research was conducted in the absence of any commercial or financial relationships that could be construed as a potential conflict of interest.
