# Supplementary material for: Lateral Preoptic Area Neurons Activated by Angiotensin-(1–7) Increase Intravesical Pressure: A Novel Feature in Central Micturition Control
Source: Front Physiol. 2021 Jul 12;12:682711. doi: 10.3389/fphys.2021.682711 (PMC8311566; doi:10.3389/fphys.2021.682711)
Supplement: Supplementary file 1 [file Presentation_1.pptx]

## Slide 1
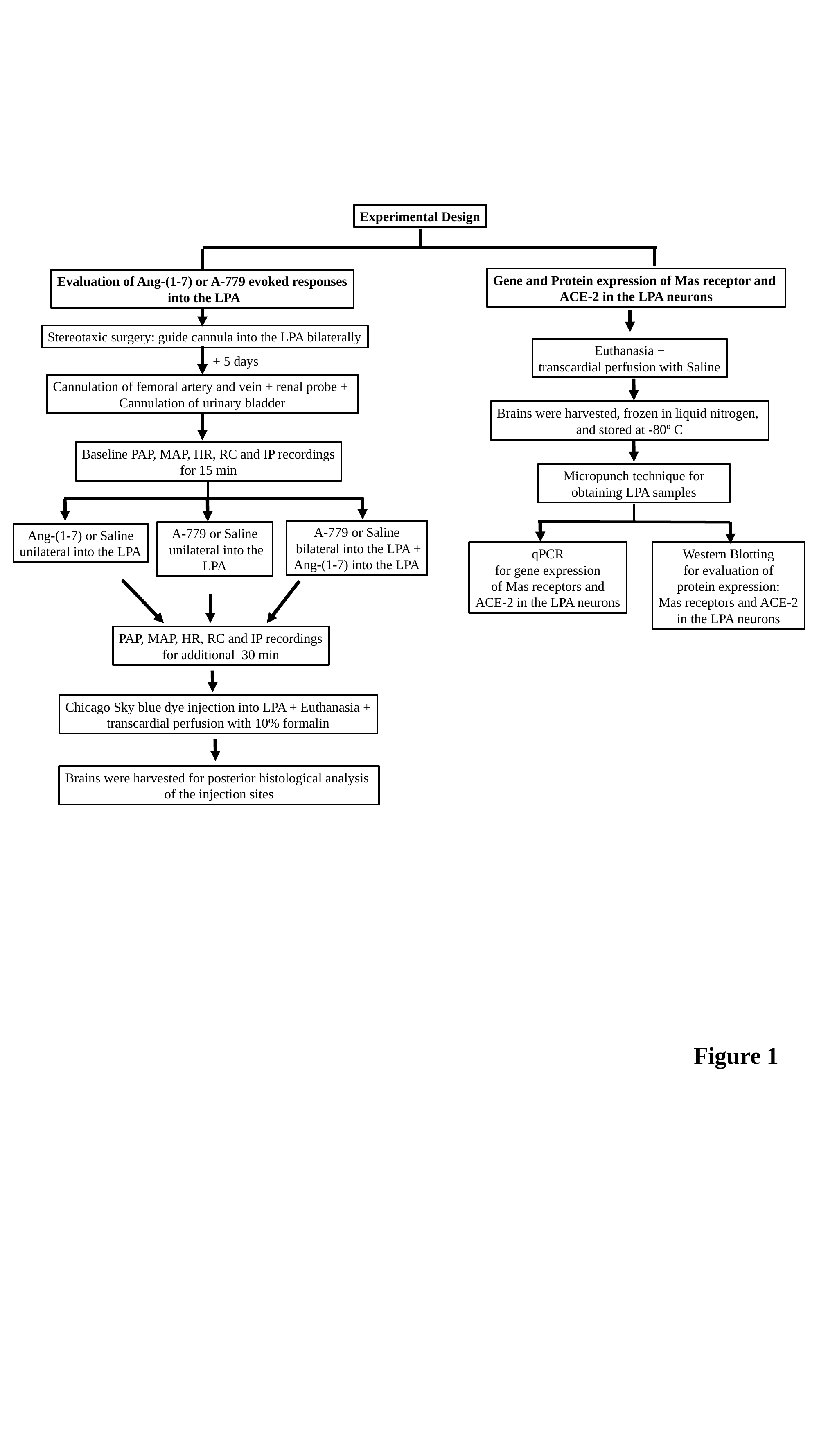

Experimental Design
Gene and Protein expression of Mas receptor and
ACE-2 in the LPA neurons
Evaluation of Ang-(1-7) or A-779 evoked responses
 into the LPA
Stereotaxic surgery: guide cannula into the LPA bilaterally
Euthanasia +
transcardial perfusion with Saline
+ 5 days
Cannulation of femoral artery and vein + renal probe +
Cannulation of urinary bladder
Brains were harvested, frozen in liquid nitrogen,
and stored at -80º C
Baseline PAP, MAP, HR, RC and IP recordings
for 15 min
Micropunch technique for obtaining LPA samples
A-779 or Saline
 bilateral into the LPA +
Ang-(1-7) into the LPA
A-779 or Saline
 unilateral into the LPA
Ang-(1-7) or Saline
unilateral into the LPA
qPCR
for gene expression
of Mas receptors and
ACE-2 in the LPA neurons
Western Blotting
for evaluation of
protein expression:
Mas receptors and ACE-2
in the LPA neurons
PAP, MAP, HR, RC and IP recordings
for additional 30 min
Chicago Sky blue dye injection into LPA + Euthanasia +
transcardial perfusion with 10% formalin
Brains were harvested for posterior histological analysis
of the injection sites
Figure 1
